# Identification of potential biomarkers for atrial fibrillation and stable coronary artery disease based on WGCNA and machine algorithms

**DOI:** 10.1186/s12872-024-04062-z

**Published:** 2024-08-02

**Authors:** Ke Wu, Hao Chen, Fan Li, Xiangjuan Meng, Lin Chen, Nannan Li

**Affiliations:** 1 Department of Cardiology, The Affiliated Taian City Central Hospital of Qingdao University, Taian, 271000 China; 2 Shandong University, Jinan, 250012 China; 3 Intensive Care Department, The Affiliated Taian City Central Hospital of Qingdao University, Taian, 271000 China; 4 Department of Interventional Radiology, The Affiliated Taian City Central Hospital of Qingdao University, Taian, 271000 China; 5 Medical Imaging Department, The Affiliated Taian City Central Hospital of Qingdao University, Taian, 271000 China; 6 Department of Respiratory and Critical Care, The Affiliated Taian City Central Hospital of Qingdao University, No. 29 Longtan Road, Taian, Shandong 27100 China

**Keywords:** Atrial fibrillation, Stable coronary artery disease, Weighted gene co-expression network analysis, Machine algorithms, Biomarkers

## Abstract

**Background:**

Patients with atrial fibrillation (AF) often have coronary artery disease (CAD), but the biological link between them remains unclear. This study aims to explore the common pathogenesis of AF and CAD and identify common biomarkers.

**Methods:**

Gene expression profiles for AF and stable CAD were downloaded from the Gene Expression Omnibus database. Overlapping genes related to both diseases were identified using weighted gene co-expression network analysis (WGCNA), followed by functional enrichment analysis. Hub genes were then identified using the machine learning algorithm. Immune cell infiltration and correlations with hub genes were explored, followed by drug predictions. Hub gene expression in AF and CAD patients was validated by real-time qPCR.

**Results:**

We obtained 28 common overlapping genes in AF and stable CAD, mainly enriched in the PI3K-Akt, ECM-receptor interaction, and relaxin signaling pathway. Two hub genes, COL6A3 and FKBP10, were positively correlated with the abundance of MDSC, plasmacytoid dendritic cells, and regulatory T cells in AF and negatively correlated with the abundance of CD56dim natural killer cells in CAD. The AUCs of COL6A3 and FKBP10 were all above or close to 0.7. Drug prediction suggested that collagenase clostridium histolyticum and ocriplasmin, which target COL6A3, may be potential drugs for AF and stable CAD. Additionally, COL6A3 and FKBP10 were upregulated in patients with AF and CAD.

**Conclusion:**

COL6A3 and FKBP10 may be key biomarkers for AF and CAD, providing new insights into the diagnosis and treatment of this disease.

**Supplementary Information:**

The online version contains supplementary material available at 10.1186/s12872-024-04062-z.

## Introduction

Cardiovascular disease, encompassing conditions like coronary artery disease (CAD) and atrial fibrillation (AF), remains the leading cause of death worldwide [1]. AF is the most prevalent serious cardiac arrhythmia, affecting 2% of the general population, with incidence increasing with age [2]. CAD, a multifactorial chronic disease, is associated with high rates of sudden death and severe complications. CAD can be categorized into stable CAD and acute coronary syndromes [3, 4].

The coexistence of AF and CAD is relatively common in clinical settings [5], with approximately 30% of patients with AF having CAD [6]. Recent research shows atherosclerotic CAD in about 70% of AF patients undergoing angiography [7]. The recurrence rate of AF was significantly reduced after percutaneous coronary intervention in patients with CAD, suggesting a direct link between AF and CAD [8]. The two diseases are likely rooted in shared risk factors and pathogenic mechanisms [9, 10]. These factors drive inflammation and endothelial dysfunction, two key elements in both diseases. Myocardial ischemia-induced local inflammation causes fibrosis and prolonged conduction time, triggering ectopic foci, re-entry, and neural alterations that may lead to AF [11]. Conversely, AF may promote the growth and instability of atherosclerotic plaques by inducing pro-inflammatory factors [12]. Additionally, the reduction of nitric oxide leads to endothelial dysfunction, increasing CAD risk [10, 13]. Therefore, it is clinically important to explore the shared underlying mechanisms of AF and CAD and identify promising biomarkers.

Machine learning has become a pivotal tool in basic and clinical research for uncovering disease mechanisms [14]. It has been employed to develop risk prediction models for AF and stable CAD [15, 16]. Genetic studies play a crucial role in understanding the mechanisms of both diseases [17, 18]. Our study aimed to identify common hub genes in AF and stable CAD that could serve as potential biomarkers, offering new insights into disease mechanisms and treatment. To achieve this, we utilized weighted gene co-expression network analysis (WGCNA) and machine learning algorithms on public databases to identify these common hub genes and explored their correlation with immune cells. Furthermore, real-time qPCR and receiver operating characteristic (ROC) curves were performed to validate the expression, diagnostic, and therapeutic values of hub genes.

## Materials and methods

### Data source and processing

Gene expression data were filtered by keywords “stable CAD” or “AF” and “Homo sapiens” using Gene Expression Omnibus (GEO) database (http://www.ncbi.nlm.nih.gov/geo/). Finally, five datasets were included. GSE159657 (plasma exosome samples, *n* = 10 for controls and *n* = 8 for stable CAD) and GSE56885 (peripheral blood mononuclear cell samples, *n* = 2 for controls and *n* = 4 for stable CAD) were in the stable CAD group. GSE79768 (thirteen paired left and right atrial tissue samples, *n* = 6 for sinus rhythm and *n* = 7 for AF), GSE115574 (59 left and right atrial tissue samples from 16 patients with sinus rhythm and 15 patients with AF), and GSE41177 (Nineteen paired left atrial-pulmonary vein junction and left atrial appendage samples, *n* = 3 for sinus rhythm and *n* = 16 for AF) were in the AF group. Merged GSE79768 and GSE115574 contained a total of 85 left and right atrial tissue samples (from 22 patients with sinus rhythm and 22 patients with AF) after removing the batch effect using the “combat” function. GSE159657 and combined GSE79768 and GSE115574 were used as the training sets, and GSE56885 and GSE41177 were used as the validation sets, respectively.

### WGCNA

GSE159657 and merged GSE79768 and GSE115574 were analyzed through the “WGCNA” package in R to obtain modules that positively related to stable CAD and AF, respectively. Genes with high expression variability (top 25% of expression standard deviation) were selected and outliers were excluded by hierarchical clustering. Scale-free networks were constructed, where connections between genes are based on their co-expression patterns. This network exhibits a power-law distribution, meaning a few highly connected genes (hub genes) influence many other genes. Appropriate thresholds (β) were selected using the “pickSoftThreshold” function to ensure the scale-free property of network (R^2^ > 0.9). Next, the topological overlap matrix (TOM) was calculated, which measures the similarity of gene connections within the network. Using dynamic tree-cutting methods, genes with high TOM values were clustered into modules, ensuring a minimum dissimilarity (phase dissimilarity < 0.25) between modules. Correlation analysis between modules’ eigenvalues (representative genes summarizing the module’s expression pattern) and clinical features of disease was performed to obtain modules related to disease. Detailed clinical information on the patients included in these datasets is described in Supplementary file 1 (Table S1-S4).

### Identification of overlapping genes and protein-protein interaction (PPI) network analysis

Intersections were taken for genes in modules positively related to AF or stable CAD to obtain overlapping genes. These overlapping genes were then imported into the STRING database (https://cn.string-db.org/) for PPI analysis (species: “Homo sapiens”) [19].

### Function enrichment analysis

To further understand the common biological mechanisms and signaling pathways between AF and stable CAD, we performed gene ontology (GO) and Kyoto Encyclopedia of Genes and Genomes (KEGG) enrichment analysis using the DAVID database (https://david.ncifcrf.gov/) with a false discovery rate (FDR) < 0.05.

### Identification of hub genes based on machine learning algorithms

Based on GSE159657 and merged GSE79768 and GSE115574, the least absolute shrinkage and selection operator (LASSO) regression analysis was performed to screen genes from the overlapping genes using the “glmnet” package in R, respectively. Screened genes were ranked by the value of the mean decrease accuracy using the “randomForest” package in R, followed by 10-fold cross-validation to obtain the optimal number of signature genes. For classification models, random forest (RF) was configured with 500 trees and enabled importance and proximity parameters in the “randomForest” package in R, while support vector machine (SVM) employed a radial basis function kernel with default settings and probability = TRUE for predicting class probabilities in the “e1071” packages in R. These parameters provided satisfactory results on the datasets. The receiver operating characteristic (ROC) curve of the classification models and signature genes was performed using the “pROC” package in R. The area under the curve (AUC) was used to evaluate their diagnostic capacity. Candidate hub genes were identified from signature genes that were differentially expressed in both the AF and stable CAD groups compared to controls. Moreover, these genes exhibited the same expression trend in both AF and stable CAD. Candidate hub genes were validated in the validation sets, and those with consistent results were confirmed as hub genes. Then, the ROC curves of the hub genes in the training and validation sets were plotted using the “pROC” package in R.

### Comprehensive analysis of hub genes

Co-expression and functional enrichment analysis of hub genes were performed on GeneMANIA (http://genemania.org). The transcription factors (TFs) regulating the hub genes were predicted using the Cistrome DB database (http://genemania.org) and those with Regulatory Potential Score in the top 20 were selected and visualized using Cytoscape to construct the “TF-mRNA” regulatory network. In addition, the DGIdb database (https://dgidb.org/) was used to predict potential drugs that may target the hub genes.

### Immune cell infiltration analysis

The relative abundance of immune cell infiltration in control and disease samples was quantified by enrichment score using the ssGSEA (Single Sample Gene Set Enrichment Analysis) algorithm, followed by comparing the differences using the t-test. Gene sets labeling each infiltrating immune cell type were obtained from Charoentong’s study [20]. Correlation analysis between pivotal genes and immune cell infiltration was conducted using Pearson correlation coefficients.

### Patient samples and protocols

A total of 28 blood samples were collected from 8 healthy controls, 6 patients with stable CAD, 6 patients with AF, and 8 patients with AF-CAD samples. Detailed clinical information on these patients is provided in Supplementary file 1 (Table S5). Total RNA from blood samples was extracted using the Blood RNA Extraction Kit (R4163-02, Magen) and reversed to cDNA using the FastQuant cDNA First-Strand Synthesis Kit (KR106, TIANGEN). Real-time PCR was performed to detect the expression levels of hub genes using SuperReal PreMix Plus (SYBR Green) (FP205, TIANGEN) according to the manufacturer’s protocol. GADPH and β-actin were used as internal reference genes. Detected data were processed using the 2^−∆∆Ct^ method. Data were shown as the means ± standard deviation (SD). T-test was used for comparison between two samples. The specific primer sequences are shown in Table 1.

**Table 1:** Primer sequences used for the real-time PCR

Primer name	Primer sequences (5’ to 3’)
GAPDH-F	GGAGCGAGATCCCTCCAAAAT
GAPDH-R	GGCTGTTGTCATACTTCTCATGG
ACTB-F	CATGTACGTTGCTATCCAGGC
ACTB-R	CTCCTTAATGTCACGCACGAT
COL6A3-F	ATGAGGAAACATCGGCACTTG
COL6A3-R	GGGCATGAGTTGTAGGAAAGC
FKBP10-F	GTGGTTCTGCTGGATGTGTG
FKBP10-R	TAGCTGGTGTCGAAGGAGGT

## Results

### Identification of modules related to AF and stable CAD

**Fig. 1:**
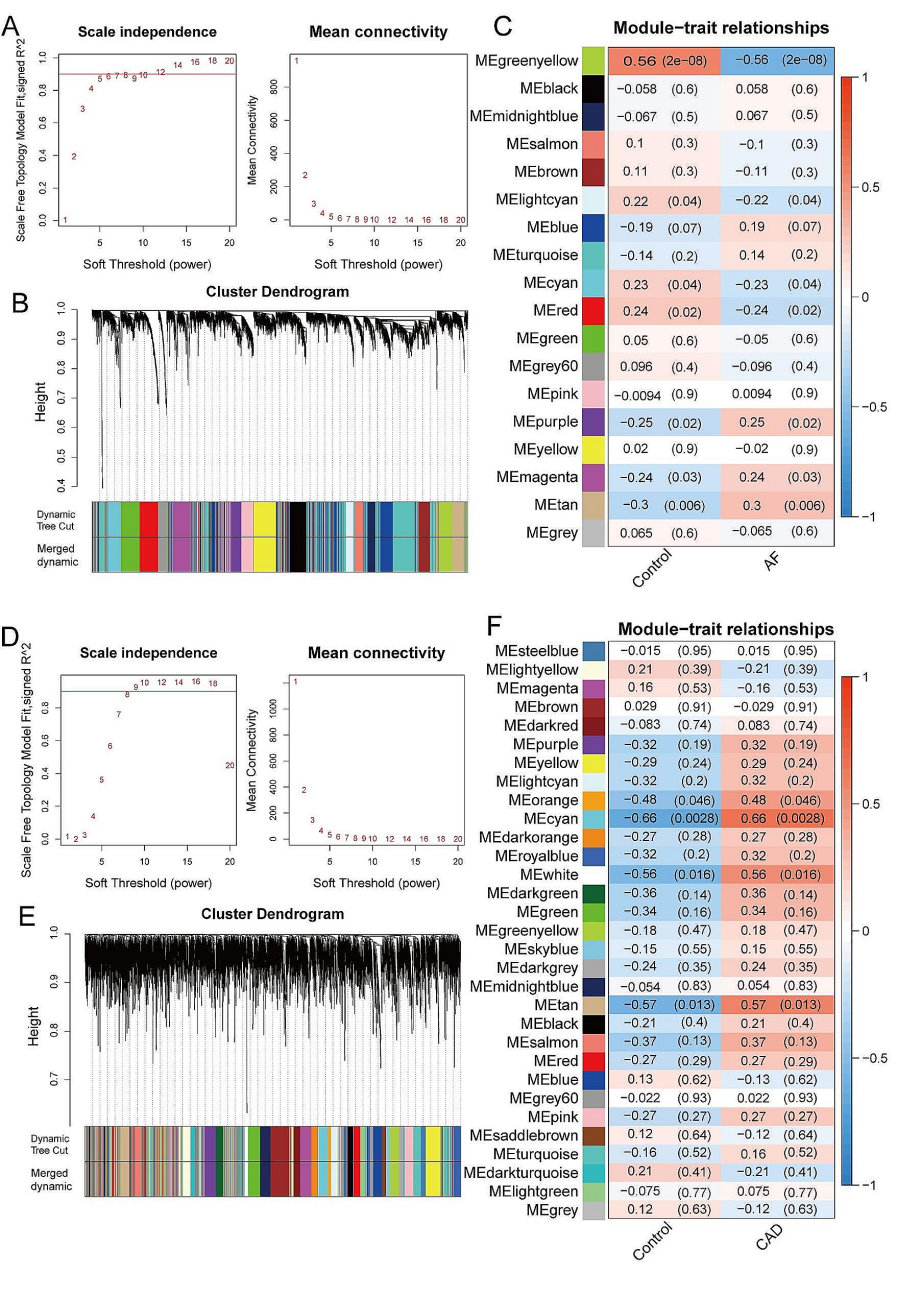
Identification of modules associated with AF and stable CAD. **A** The scale-free fit index for soft-thresholding powers and mean connectivity (combined GSE79768 and GSE115574). **B** Gene dendrogram and module colors. **C** Correlation results between the 18 modules and AF trait. **D** The scale-free fit index for soft-thresholding powers and mean connectivity (GSE159657). **E** Gene dendrogram and module colors. **F** Correlation results between the 31 modules and stable CAD trait

A total of 21,665 common genes in merged GSE79768 and GSE115574 were obtained by removing batch effects using the “combat” function. The data distributions between the two datasets converge after batch effect removal, indicating effective mitigation of batch effects (Figure S1). In merged GSE79768 and GSE115574, β = 5 was chosen using the “pickSoftThreshold” function to construct scale-free networks (Fig. 1A). The minimum number of genes in a module was set to 100 and 18 modules were obtained after cutting the dynamic tree (Fig. 1B). Correction analysis revealed that 3 modules were significantly and positively related to AF, including purple (*r* = 0.25), magenta (*r* = 0.24), and tan (*r* = 0.3) modules (Fig. 1C). In GSE159657, β = 8 was chosen using the “pickSoftThreshold” function to construct scale-free networks (Fig. 1D). The minimum number of genes in a module was set to 40 and 31 modules were obtained after cutting the dynamic tree (Fig. 1E). Correction analysis revealed that 4 modules were significantly and positively related to stable CAD, including orange (*r* = 0.48), cyan (*r* = 0.66), white (*r* = 0.56), and tan (*r* = 0.57) modules (Fig. 1F).

### Identification of overlapping genes

**Fig. 2:**
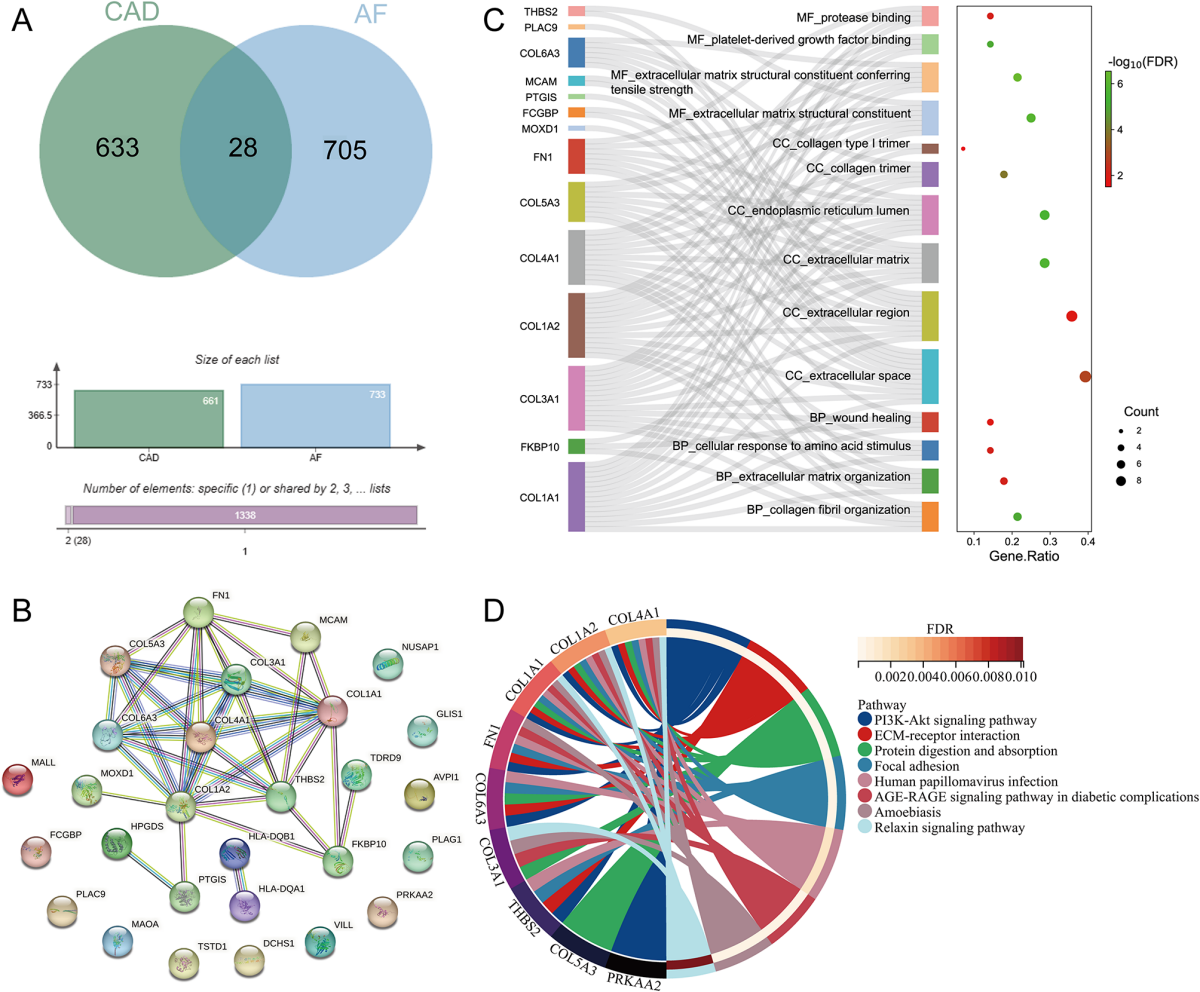
Identification of overlapping genes and functional enrichment. **A** Venn diagram of CAD and AF module genes. **B** PPI network of overlapping genes. **C** GO enrichment analysis of overlapping genes. **D** KEGG enrichment analysis of overlapping genes

The genes in modules related to AF and stable CAD were intersected to obtain 28 overlapping genes (Fig. 2A). The PPI network of overlapping genes identified 28 pairs of interacting genes. Among them, 4 pairs of genes with interaction scores were at 0.999, including COL1A1 and COL3A1, COL1A1 and COL1A2, COL1A2 and COL3A1, HLA-DQA1 and HLA-DQB1 (Fig. 2B). GO analysis showed that those overlapping genes were mainly distributed in the cellular component (CC) such as extracellular space and collagen trimer. These genes were mainly enriched in molecular functions (MF) such as extracellular matrix structural constituent, protease binding, and platelet-derived growth factor binding. These genes were mainly involved in biological processes (BP) such as collagen fibril and extracellular matrix organizations, cellular responses to amino acid stimuli, and wound healing (Fig. 2C). KEGG analysis showed that these genes were enriched in PI3K-Akt, relaxin Signaling Pathway, Extra Cellular Matrix (ECM)-receptor interaction, and focal adhesion (Fig. 2D).

### Identification of hub genes based on the machine learning algorithm

**Fig. 3:**
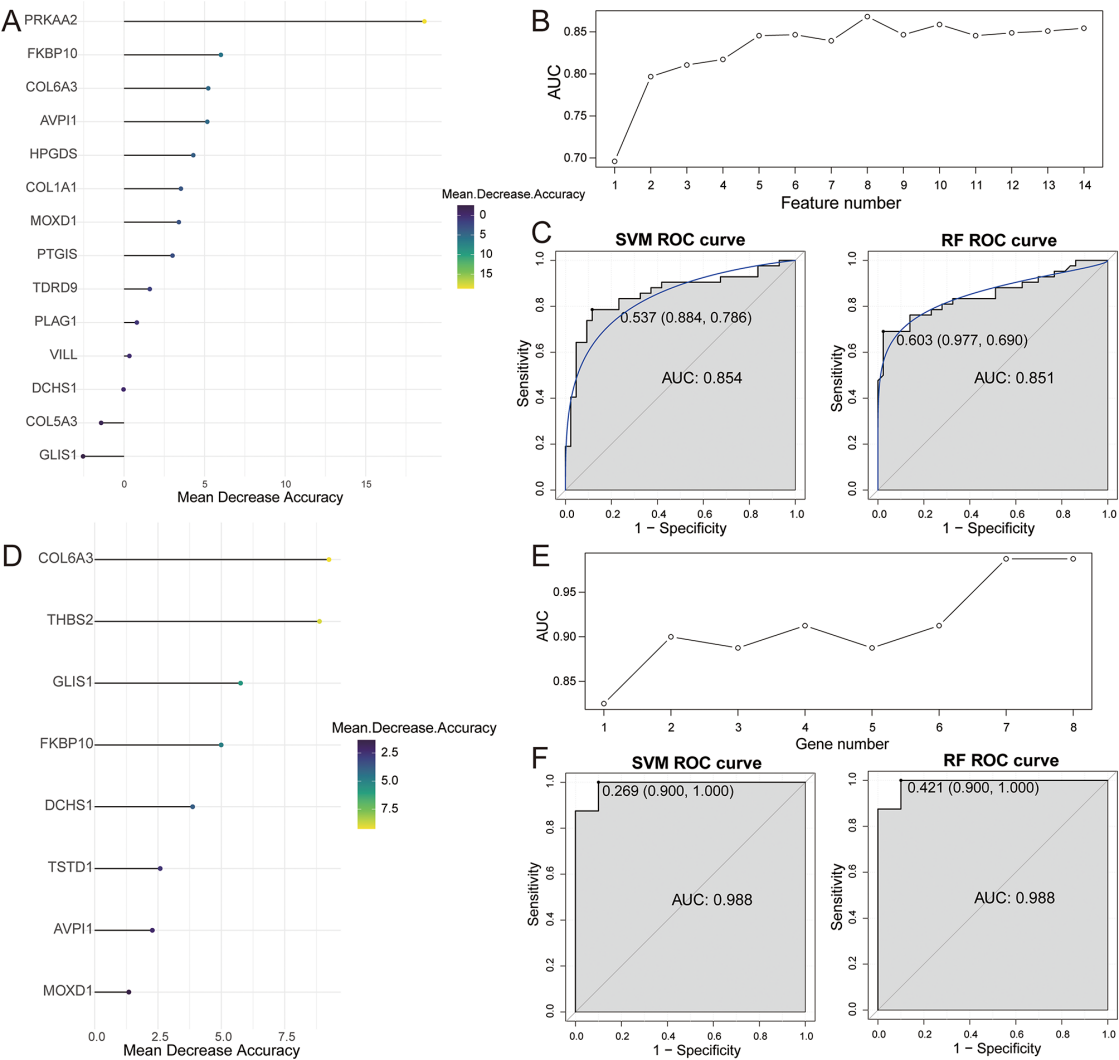
Identification of candidate hub genes based on machine learning algorithm. **A** Genes screened by LASSO and ranked by average decreasing precision in AF dataset. **B** The results of 10-fold cross validation. **C** ROC curves for model prediction accuracy in AF dataset. **D** Genes screened by LASSO and ranked by average decreasing precision in CAD dataset. **E** The results of 10-fold cross validation. **F** ROC curves for model prediction accuracy in CAD dataset

Diagnostic models for AF and CAD were constructed through machine learning algorithms. In merged GSE79768 and GSE115574, 14 genes were screened from 28 overlapping genes using LASSO and ranked by mean decrease accuracy (Fig. 3A). The 10-fold cross-validation showed that the AUC of diagnosing AF was highest when the number of genes was 8 (Fig. 3B). Subsequently, SVM and RF models of AF were constructed using 8 signature genes. The AUCs of SVM and RF were 0.854 and 0.851, respectively (Fig. 3C). The SVM model achieved an accuracy of 0.812, a sensitivity of 0.786, and a specificity of 0.884. The RF model showed an accuracy of 0.800, a sensitivity of 0.690, and a specificity of 0.977. Similarly, 8 genes were screened in GSE159657 (Fig. 3D) and the AUC of diagnosing stable CAD was highest when the number of genes was 7 (Fig. 3E). SVM and RF models of stable CAD were also constructed using 7 signature genes. The AUCs of both the SVM and RF were 0.988 (Fig. 3F). The SVM model had an accuracy of 0.889, a sensitivity of 1.000, and a specificity of 0.900. The RF model exhibited comparable performance metrics to the SVM.

**Fig. 4:**
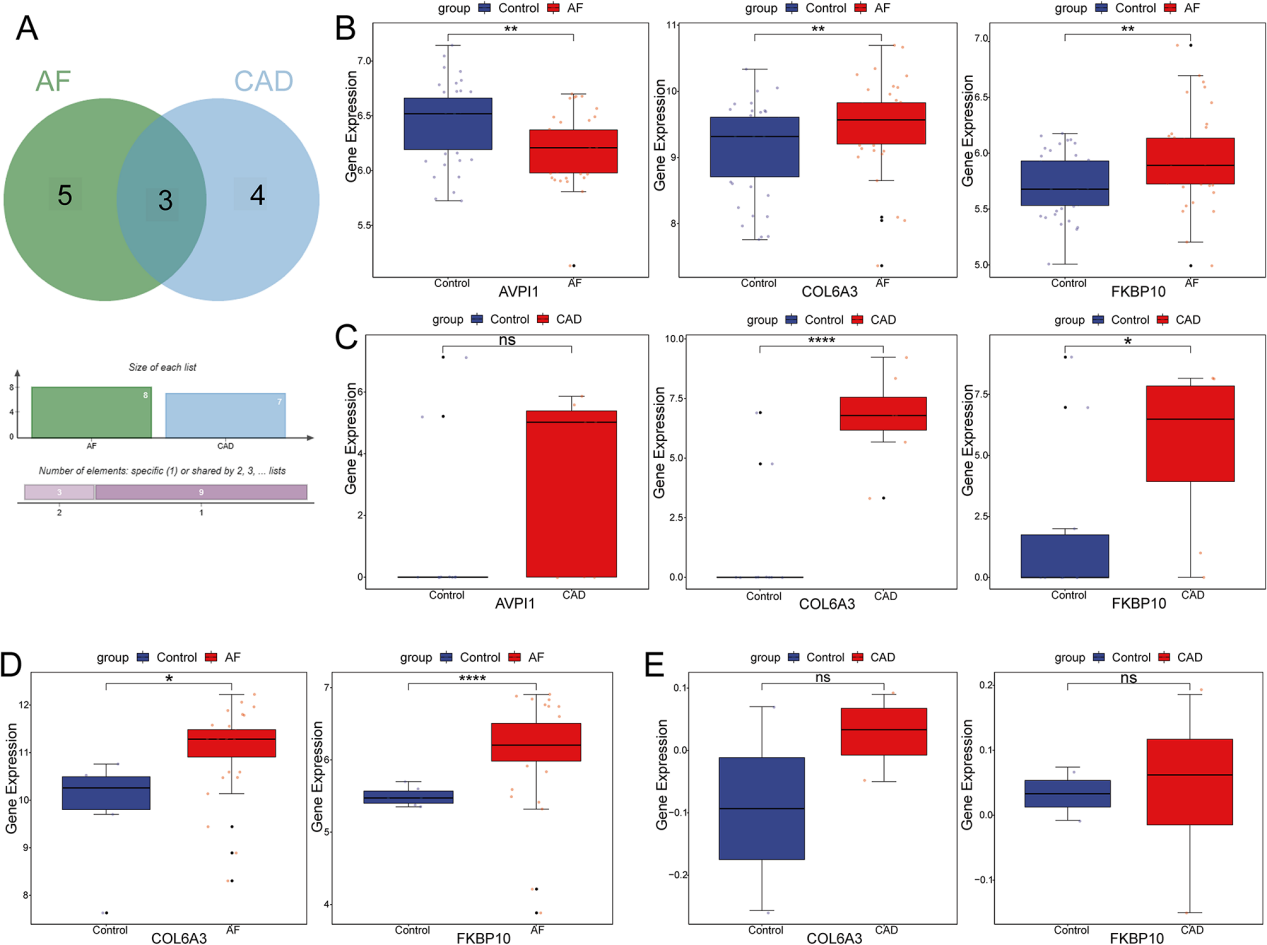
The expressions of candidate genes were verified in training and validation sets. **A** Venn diagram of AF and CAD related genes. **B** The expression of candidate genes of AF in training sets. **C** The expression of candidate genes of CAD in training sets. **D** The expression of candidate genes of AF in validation sets. **E** The expression of candidate genes of CAD in validation sets

Additionally, 8 AF-related signature genes and 7 stable CAD-related signature genes were overlapped to obtain 3 genes (COL6A3, FKBP10, and AVPI1) (Fig. 4A). The expression levels of COL6A3 and FKBP10 in training sets were significantly highly expressed in both AF and stable CAD, while AVPI1 expression was inconsistent (Fig. 4B-C). Therefore, COL6A3 and FKBP10 were used as candidate hub genes and further validated for expression in the validation sets. Validation showed that COL6A3 and FKBP10 were significantly up-regulated in AF in the GSE41177 dataset and trended towards high expression in stable CAD in the GSE56885 dataset (Fig. 4D-E) Finally, COL6A3 and FKBP10 were identified as hub genes potentially associated with stable CAD and AF.

### Comprehensive analysis of hub genes

**Fig. 5:**
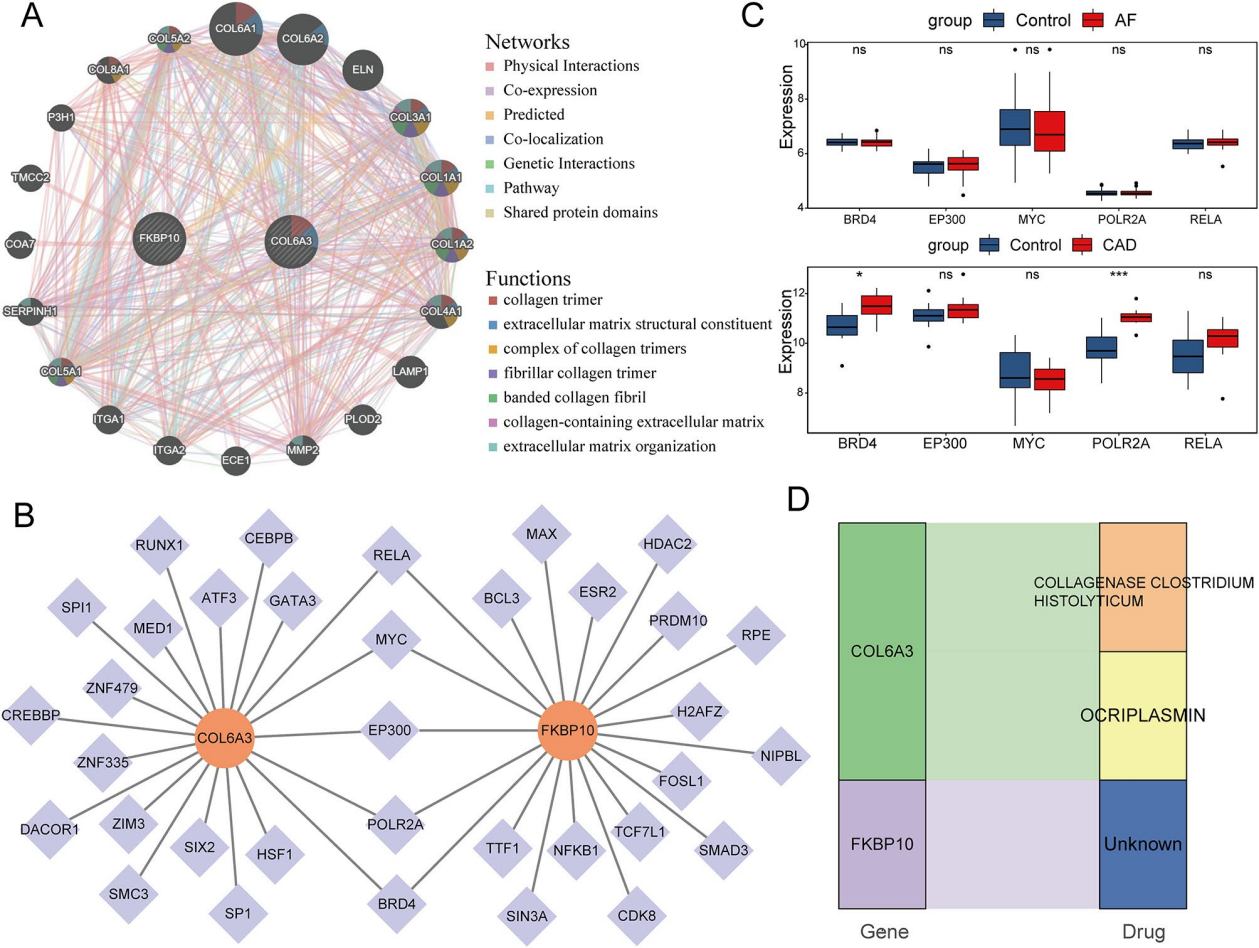
Comprehensive analysis of hub genes. **A** Co-expression network of COL6A3 and FKBP10, and functional enrichment. **B** The “TF-mRNA” regulatory network of COL6A3 and FKBP10. **C** The expression of common transcription factors in AF and CAD. **D** Drug prediction for COL6A3 and FKBP10 based on DIGDB database

A complex network was constructed using GeneMANIA, including 20 genes related to COL6A3 and FKBP10, and 778 interactions (Fig. 5A). The functional enrichment results suggested that these 22 genes may be linked to various aspects of the extracellular matrix, such as collagen trimer (Fig. 5A). The “TF-mRNA” regulatory network revealed 5 common TFs (BRD4, EP300, MYC, POLR2A, and RELA) that regulate COL6A3 and FKBP10 (Fig. 5B). The expression levels of BRD4 and POLR2A were significantly higher in the stable CAD group compared to the controls, while there was no significant difference between the AF group and its controls (Fig. 5C). Therefore, it is hypothesized that hub genes in CAD could be regulated by BRD4 and POLR2A, while in AF they may be regulated by other TFs. Furthermore, the drug prediction results revealed that two drugs targeted COL6A3, including collagenase clostridium histolyticum and ocriplasmin, while there were no corresponding drugs searched for FKBP10 (Fig. 5D).

### Assessment of the predictive accuracy of hub genes

**Fig. 6:**
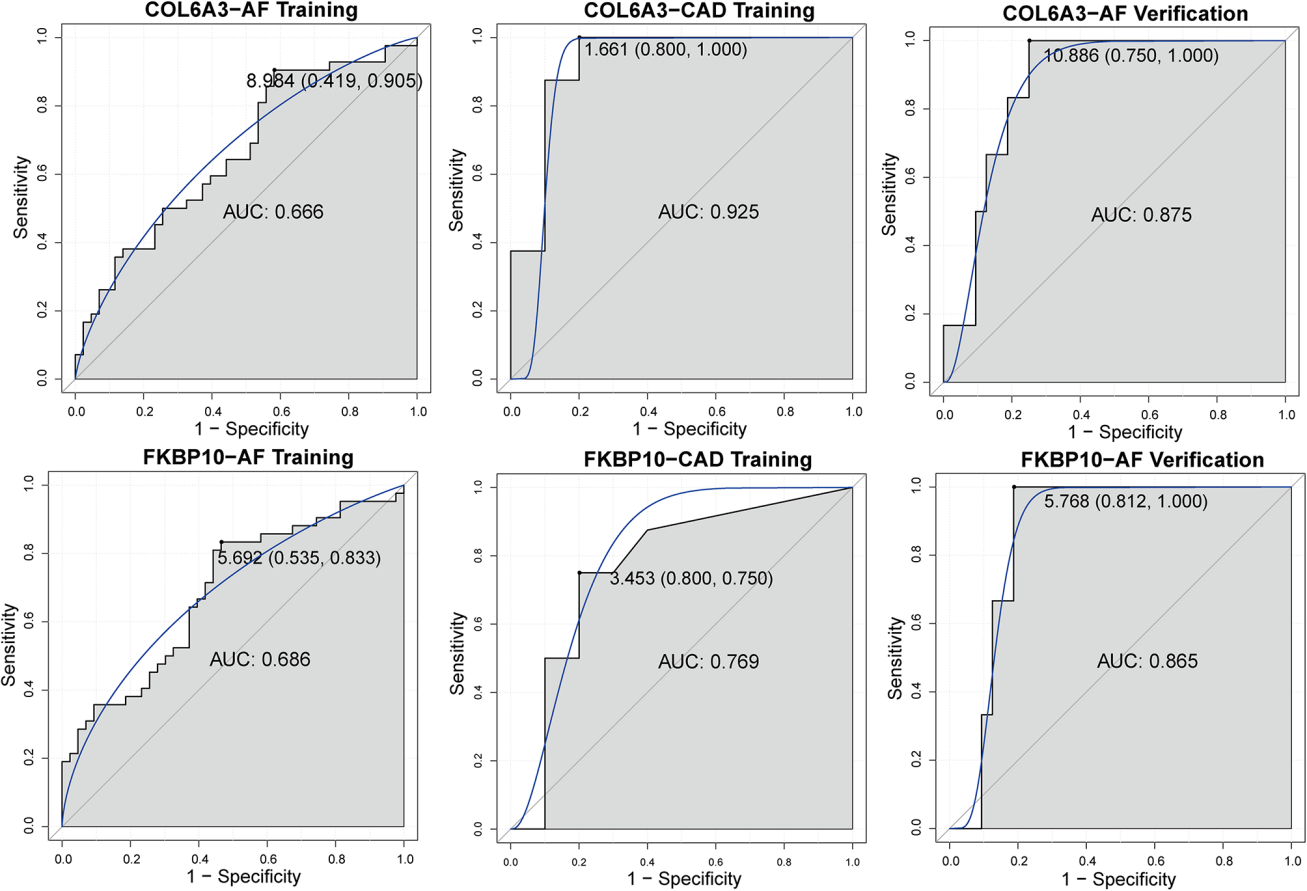
ROC curve of hub genes in training and validation sets

To evaluate the diagnostic ability of hub genes for two diseases, we performed ROC analysis. The validation set for CAD was excluded from this analysis due to its small sample size. The results showed that the AUC values were consistently near or exceeded 0.7 in all three datasets, suggesting the potential ability of COL6A3 and FKBP10 to distinguish patients with AF and CAD from controls (Fig. 6).

### Immune cell infiltration analysis

**Fig. 7:**
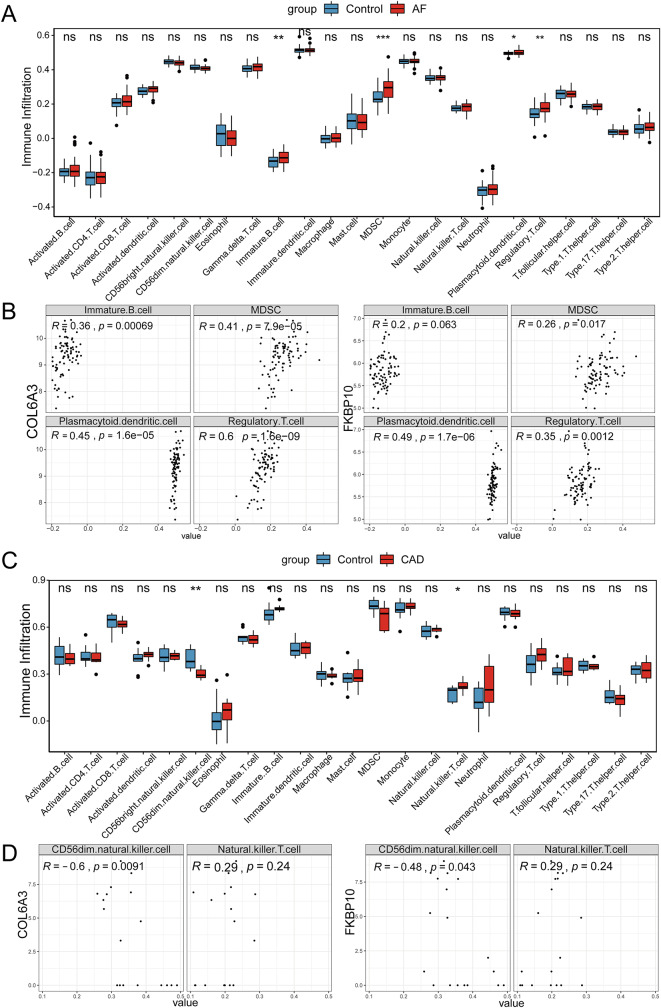
Analysis of immune cell infiltration. **A** The comparison of immune infiltration in the AF and control group. **B** Correlation analysis between differential immune cell in AF with COL6A3 and FKBP10. **C** The comparison of immune infiltration in the CAD and control group. **D** Correlation analysis between differential immune cell in CAD with COL6A3 and FKBP10

Given the pathogenic role of inflammation in both stable CAD and AF, we performed immune cell infiltration analyses in training sets for the two diseases. The results showed that the levels of immature B cells, myeloid-derived suppressor cells (MDSC), plasmacytoid dendritic cells, and regulatory T cells were significantly higher in the AF group compared to the controls (Fig. 7A). Correlation analysis showed that COL6A3 was significantly positively correlated with immature B cells (*r* = 0.36), MDSC (*r* = 0.41), plasmacytoid dendritic cells (*r* = 0.45), and regulatory T cells (*r* = 0.6). FKBP10 showed significant positive correlations with MDSC (*r* = 0.26), plasmacytoid dendritic cells (*r* = 0.49), and regulatory T cells (*r* = 0.35), but not with immature B cells (Fig. 7B). Similarly, the levels of CD56dim natural killer cells were significantly decreased in the stable CAD group compared to the controls, while natural killer T cells significantly increased (Fig. 7C). Meanwhile, CD56dim natural killer cells exhibited significant negative correlations with COL6A3 (*r* = -0.6) and FKBP10 (*r* = -0.48). However, no significant correlation was found between natural killer T cells and the two hub genes (Fig. 7D).

### Validation of hub genes in patients with stable CAD and AF

**Fig. 8:**
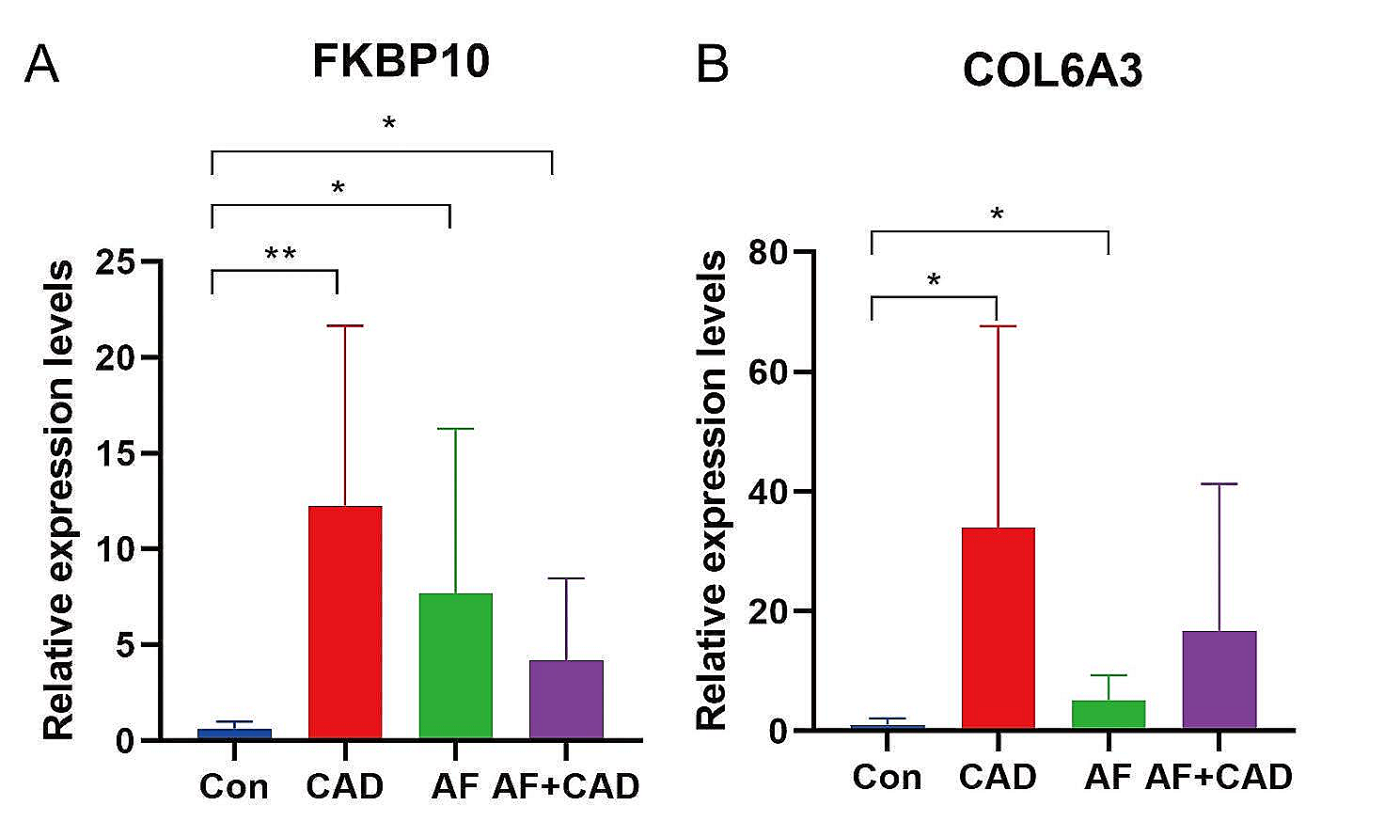
Validation of hub genes in patients with stable CAD and AF

The expression levels of FKBP10 were significantly higher in all groups (CAD, AF, and AF + CAD) compared to controls (Fig. 8A). The expression levels of COL6A3 were significantly higher in the CAD and AF groups compared to controls (Fig. 8B). Although the AF + CAD group did not show a significant difference from the control group, there was a trend toward increased expression.

## Discussion

A two-sample Mendelian randomization study showed that CAD is an independent risk factor for AF [21], suggesting a strong relationship between the two diseases. Another study showed a greater correlation between D-dimer and CAD in patients with AF compared to non-AF patients [22]. However, the shared mechanism of stable CAD and AF remains unclear. In this study, we first analyzed genetic datasets from both diseases to uncover common mechanisms and key biomarkers.

Twenty-eight overlapping genes were identified through WGCNA and positively associated with AF and stable CAD. These genes may be associated with collagen fibers, extracellular matrix structure, and wound healing. Atrial tissue fibrosis is an important feature of AF [23]. Histological studies show that AF-associated atrial remodeling manifests as fibrosis and enlarged myogenic fibers [24]. Wound healing is significant in the prognosis of myocardial infarction and affects the outcome of catheter ablation for AF, potentially causing serious complications [25, 26]. KEGG analysis revealed that these overlapping genes were significantly enriched in PI3K-Akt, ECM-receptor interaction, and relaxin signaling pathways. ECM deposition is a major pathological feature of AF [27]. PI3K-Akt could regulate cardiac fibroblast proliferation and ECM deposition [27], while ECM alterations are linked to inflammation and plaque formation in atherosclerosis [28]. Relaxin can cause vasorelaxation by modulating endothelium-dependent responses and is clinically evaluated for CAD treatment [29], also having anti-inflammatory and antifibrotic effects beneficial for AF patients [30]. Therefore, functional annotation suggests these overlapping genes may contribute to the pathology of AF and CAD.

Two hub genes, COL6A3 and FKBP10, were further identified by a machine learning algorithm as significantly up-regulated in both the AF and CAD groups. COL6A3 encodes collagen VI, involved in connective tissues development and aorta tensile strength [31]. It may regulate cardiovascular dysfunction in CAD patients through aging-related genes [32]. FKBP10, a chaperone protein, interacts with collagen I [33] and may be targeted by SHOX2 to promote conduction traits in AF [34]. FKBP10 also interacts with collagen VI and co-regulates lung fibroblast migration [30]. These previous studies demonstrate that COL6A3 and FKBP10 are key regulators of ECM remodeling, playing important roles in AF and CAD development. Nonetheless, their specific roles in AF and stable CAD remain to be elucidated. The DGIdb database predicted potential therapeutic drugs for AF and CAD, such as collagenase clostridium histolyticum for fibrotic plaque-like disease [35] and Ocriplasmin for ophthalmic diseases [36]. The ROC curve results implied that COL6A3 and FKBP10 have good diagnostic ability for AF and stable CAD, and real-time PCR results confirmed the bioinformatics results.

We constructed a “TF-mRNA” regulatory network to understand the possible regulatory relationship between COL6A3 and FKBP10 in AF and CAD. BRD4 and POLR2A may regulate these genes in CAD. Increased BRD4 expression in coronary arteries of pulmonary hypertension patients contributes to vascular remodeling [37]. Although BRD4 expression did not differ between AF and controls, it is important in cardiac fibrosis [38]. POLR2A is upregulated in Kawasaki disease patients and may be associated with coronary artery abnormalities [39]. Despite no difference in POLR2A between AF and controls, it is a potentially stable internal reference gene in different cardiac cavities and disease conditions [40].

Inflammation is a common feature of AF and CAD, and the incidence of these conditions appears to increase with inflammation [41]. Patients with AF often exhibit upregulation of platelet-binding factor and plasma stromal cell-derived factor 1, which are risk factors for CAD and play roles in immune cell recruitment [42]. Targeting inflammation through treatments may help prevent the development of AF or reduce its recurrence in patients with CAD [11]. It has shown that gut microbes may control inflammation in elderly AF patients by regulating regulatory T cells [43], and CAD patients have lower levels of CD56dim natural killer cells [44]. In some diseases, COL6A3 is associated with immune responses and may be regulated by the PI3K, Jun, and NF-κB pathways [45, 46]. FKBP proteins modulate immune responses with immunosuppressive drugs [47]. Combined with previous studies, it is hypothesized that COL6A3 and FKBP10 may influence the development of AF and stable CAD through immune cell infiltration.

This study has some limitations. The limited size of the dataset could potentially bias the results, especially the validation set GSE56885 for CAD, which likely represents the principal restriction of the study. Furthermore, these findings are based on public databases and require validation in larger clinical cohorts and experimental models.

## Conclusion

Our study delved into the genetic basis and common mechanisms between AF and CAD. We identified COL6A3 and FKBP10 as significantly upregulated in both AF and stable CAD, with good diagnostic abilities and potential as therapeutic targets. These findings enhance our understanding of the shared mechanisms and offer promising insights into the treatment of these two diseases.

### Electronic supplementary material

Below is the link to the electronic supplementary material.

Supplementary Material 1

Supplementary Material 2: Figure S1 Batch effect removal in merged GSE79768 and GSE115574 datasets for AF. (A) Box plot showing sample distribution before and (D) after batch effect removal. (B) Density plot illustrating sample distribution before (top) and after (bottom) batch effect removal. (C) UMAP plot demonstrates that samples from the two datasets cluster separately before batch effect removal (top) and intermingle after batch effect removal (bottom).

## Data Availability

The datasets used and/or analysed during the current study are available from the corresponding author on reasonable request.

## Abbreviations

AF: atrial fibrillation
CAD: coronary artery disease
WGCNA: weighted gene co-expression network analysis
GEO: Gene Expression Omnibus
TOM: topological overlap matrix
GO: gene ontology
KEGG: Kyoto Encyclopedia of Genes and Genomes
FDR: false discovery rate
LASSO: least absolute shrinkage and selection operator
RF: random forest
SVM: support vector machine
ROC: receiver operating characteristic
AUC: area under the curve
TFs: transcription factors
ssGSEA: Single Sample Gene Set Enrichment Analysis
SD: standard deviation
CC: cellular component
MF: molecular functions
BP: biological processes
ECM: Extra Cellular Matrix
MDSC: myeloid-derived suppressor cells
