# Supplementary material for: Identification of potential biomarkers for atrial fibrillation and stable coronary artery disease based on WGCNA and machine algorithms
Source: BMC Cardiovasc Disord. 2024 Aug 2;24:401. doi: 10.1186/s12872-024-04062-z (PMC11295489; doi:10.1186/s12872-024-04062-z)
Supplement: Supplementary file 1 — Supplementary Material 1 [file 12872_2024_4062_MOESM1_ESM.docx]

**Table S1 Clinical baseline characteristics of patients in GSE159657 dataset (PMID: 34490374)**

|  | **CAD (*n* = 8)** | **Control (*n* = 10)** |
| --- | --- | --- |
| **Gender** |  |  |
| Male | 7 | 2 |
| Female | 1 | 8 |
| Age (years) | 53.88 ± 9.52 | 47.00 ± 14.02 |
| Diabetes | 3 | 0 |
| **Hypertension** |  |  |
| Grade 1 | 0 | 0 |
| Grade 2 | 2 | 0 |
| Grade 3 | 4 | 0 |
| Current smoking | 0 | 0 |
| Current drinking | 0 | 0 |
| Absolute monocyte count | 0.55 ± 0.26 | 0.44 ± 0.09 |
| The percentage of monocyte | 8.29 ± 2.42 | 7.24 ± 0.78 |
| Absolute neutrophil count | 4.03 ± 1.80 | 3.96 ± 1.90 |
| The percentage of neutrophil | 59.88 ± 8.01 | 59.58 ± 11.48 |
| Creatine kinase, MB Form (U/L) | 11.80 ± 2.21 | 10.4 ± 0.64 |
| Hydroxybutyrate-dehydrogenase (U/L) | 89.00 ± 12.29 | 97.30 ± 13.21 |

**Table S2 Clinical baseline characteristics of patients in GSE79768 dataset (PMID: 27494721)**

|  | **SR (*n* = 6)** | **AF (*n* = 7)** |
| --- | --- | --- |
| **Gender** |  |  |
| Male | 2 | 3 |
| Female | 4 | 4 |
| Age (years) | 63.83 ± 20.69 | 48.29 ± 10.98 |
| **Clinical features** |  |  |
| Ejection fraction(%) | 51.83 ± 20.69 | 60.29 ± 11.35 |
| Left arterial diameter(mm) | 47.83 ± 10.54 | 59.43 ± 9.87 |
| Rheumatic heart disease | 0 | 5* |
| Hypertension | 3 | 1 |
| Diabetes mellitus | 2 | 0 |
| Coronary artery disease | 2 | 0 |

^⁎^ *P* < 0.05 versus SR

**Table S3 Clinical baseline characteristics of patients in GSE115574 dataset (PMID: 34737791)**

| **Characteristic** | **AF (*n* = 15)** | **SR (*n* = 16)** | ***P* value** |
| --- | --- | --- | --- |
| Age, years, mean ± SD | 67.0 ± 13.0 (28-80) | 59.3 ± 11.6 (33-82) | 0.098 |
| Sex, male, *n* (%) | 4 (26.6) | 5 (33.3) | 0.8 |
| Body surface area, m^2^, (min-max) | 1.81 ± 0.17 (1.39-2.05) | 1.87 ± 0.17 (1.66-2.31) | 0.341 |
| **Medical history** | | | |
| Smoking, *n* (%) | 7 (46.6) | 8 (53.3) | 0.9 |
| COAD, *n* (%) | 5 (33.3) | 4 (26.6) | 0.8 |
| Diabetes mellitus, *n* (%) | 3 (20) | 4 (26.6) | 0.20 |
| CVD, *n* (%) | 4 (26.6) | 1 (6.6) | 0.134 |
| Hypertension, *n* (%) | 11 (73.3) | 4 (26.6) | **0.015** |
| Hyperlipidemia, *n* (%) | 8 (53.3) | 5 (33.3) | 0.148 |
| Total cholesterol, mg/dl | 185.8 ± 7.1 | 185.8 ± 7.6 | 0.999 |
| LDL cholesterol, mg/dl | 116.5 ± 5.6 | 115.7 ± 6.3 | 0.93 |
| HDL cholesterol, mg/dl | 44.4 ± 4.2 | 39.5 ± 1.9 | 0.35 |
| LVEDD, mm | 54.6 ± 6.9 | 54.3 ± 12 | 0.927 |
| LVESD, mm | 34.7 ± 6.2 | 35.5 ± 9.4 | 0.748 |
| DBP, mmHg | 86.8 ± 11.4 | 89 ± 9.6 | 0.07 |
| LVEF, % | 52.9 ± 8.9 | 53.0 ± 7.1 | 0.964 |
| Systolic PAP, mmHg | 54.7 ± 9 | 43 ± 16 | **0.008** |
| LA diameter, mm | 55.9 ± 6.2 | 47.7 ± 6.3 | **<0.0001** |
| NYHA, > class 2, *n* (%) | 10 (66.6) | 9 (60) | 0.3 |
| Tricuspid regurgitation, > 2, *n* (%) | 6 (40) | 2 (13.3) | **<0.0001** |
| Euroscore, mean ± SD | 5.8 ± 3.5 | 3.8 ± 2.4 | 0.051 |
| Logistic Euroscore, % | 8.2 ± 8.1 | 4.1 ± 4.8 | 0.07 |
| **Medications** | | | |
| Antihyperlipidemic, *n* (%) | 7 (46.6) | 7 (46.6) | 1 |
| Digitalis, *n* (%) | 5 (33.3) | 2 (13.3) | 0.184 |
| Calcium channel blocker, *n* (%) | 5 (33.3) | 0 (0) | **0.014** |
| Beta blocker, *n* (%) | 11 (73.3) | 10 (66.6) | 0.621 |
| ACE/ARB inhibitors, *n* (%) | 9 (60) | 8 (53.3) | 0.224 |

Data presented as mean ± SD (min-max). *P* < 0.05 Student's *t*-test for paired comparisons. AF: atrial fibrillation; SR: sinus rhythm; BSA: body surface area; COPD: chronic obstructive pulmonary disease; CVD: cerebrovascular disease; LVEDD: left ventricular end-diastolic diameter; LVESD: left ventricular end-systolic diameter; DBP: diastolic blood pressure; LVEF: left ventricular ejection fraction; PAP: pulmonary artery pressure; LA: left atrium; NYHA: New York Heart Association; ACE: angiotensin-converting enzyme; ARB: angiotensin II receptor blockers.

**Table S4 Clinical characteristics of patients in GSE41177 dataset (PMID: 23183193)**

|  | **Sex** | **Age (y)** | **AF duration (mo)** | **Valvular replacement indication** | **LVEF (%)** | **Ischemic heart disease** | **Hypertension** | **Diabetes**  **mellitus** | **Left atrial**  **diameter**  **(mm)** | **Treatment** |
| --- | --- | --- | --- | --- | --- | --- | --- | --- | --- | --- |
| **AF1** | M | 61 | 15 | MR | 28 | + | + | - | 54 | DG, BB, DI, SP |
| **AF2** | F | 47 | 72 | MS | 60 | + | - | - | 58 | DG |
| **AF3** | M | 60 | 102 | AS | 60 | - | - | - | 4 | ARB, DI |
| **AF4** | F | 71 | 48 | MS, AS | 60 | - | - | - | 61 | DG, DI, CCB |
| **AF5** | F | 32 | 10 | MS | 64 | - | - | - | 66 | DG, DI |
| **AF6** | M | 64 | >1 | MR | 34 | + | + | + | 41 | BB, DI, ARB |
| **AF7** | F | 32 | 14 | MR | 72 | - | - | - | 52 | AM, ACEI, DI, DG |
| **AF8** | M | 43 | 150 | MS, AS | 77 | - | - | - | 59 | DG, DI |
| **AF9** | F | 65 | 10 | MR | 34 | - | - | - | 56 | BB, DI |
| **AF10** | M | 56 | 100 | MR | 67 | - | - | - | 51 | BB, DI, DG |
| **AF11** | M | 66 | 78 | MR | 66 | - | + | + | 50 | DG, DI, ARB, BB, CCB |
| **AF12** | M | 36 | >1 | MS, MR | 60 | - | - | - | 45 | DI, ARB, AM |
| **AF13** | M | 51 | >1 | MR | 65 | - | + | - | 53 | BB, DI |
| **AF14** | F | 65 | 110 | MS | 73 | - | - | - | 59 | DG, SP |
| **AF15** | F | 59 | >1 | MS | 65 | - | - | + | 43 | DG, CCB |
| **AF16** | F | 59 | 73 | MS | 69 | - | - | - | 65 | DG |
| **SR1** | F | 62 | - | MR | 21 | + | + | + | 67 | ARB, BB, DI |
| **SR2** | M | 43 | - | MR | 66 | - | + | + | 42 | ARB, DI |
| **SR3** | M | 55 | - | MR | 79 | - | - | - | 36 | DI |

AM ¼ amiodarone; ARB ¼ angiotensin II receptor blocker; AS ¼ aortic stenosis; BB ¼ beta blocker; CCB ¼ calcium channel blocker; DG ¼ digitalis; DI ¼

diuretics; F ¼ female; LVEF ¼ left ventricular ejection fraction; M ¼ male; MR ¼ mitral regurgitation; MS ¼ mitral stenosis; SP ¼ spironolactone.

Please note: Clinical baseline characteristics of patients in GSE56885 dataset were not found.

**Table S5 Clinical characteristics of patients with atrial fibrillation and stable coronary artery disease in the current study**

|  | **Control (*n* = 8)** | **CAD (*n* = 6)** | **AF (*n* = 6)** | **AF+CAD (*n* = 8)** |
| --- | --- | --- | --- | --- |
| **Gender** |  |  |  |  |
| Male | 4 | 1 | 1 | 5 |
| Female | 4 | 5 | 5 | 3 |
| Age (years) | 45.88 ± 16.23 | 60.5 ±9.61 | 62.5 ± 9.40 | 73.88 ± 7.47 |
| BMI | 26.46 ± 3.26 | 24.82 ± 2.86 | 27.13 ± 4.81 | 23.49 ± 2.36 |
| Heart rhythm | 69.13 ± 11.18 | 69 ± 11.03 | 79.67 ± 20.80 | 99.5 ± 21.08 |
| Diabetes | 0 | 0 | 0 | 0 |
| Hypertension | 4 | 2 | 1 | 4 |
| Dyslipidemia | 2 | 3 | 2 | 1 |
| Smoking | 1 | 3 | 1 | 1 |
| Drinking | 1 | 4 | 1 | 0 |
| Absolute monocyte count | 0.47 ± 0.22 | 0.51 ± 0.18 | 0.53 ± 0.19 | 0.49 ± 0.16 |
| Absolute neutrophil count | 3.43 ± 1.16 | 3.07 ± 1.06 | 3.36 ± 0.43 | 3.16 ± 1.29 |
| Creatine kinase, MB Form (U/L) | 1.27 ± 0.48 | 2.81 ± 2.22 | 1.75 ± 1.15 | 1.49 ± 0.59 |
| Hydroxybutyrate-dehydrogenase (U/L) | 132.25 ± 31.57 | 150.67 ± 33.55 | 148.33 ± 24.31 | 174.63 ± 42.99 |

Data were presented as mean ± SD.
