# Supplementary figures and images for: Identification of potential biomarkers for atrial fibrillation and stable coronary artery disease based on WGCNA and machine algorithms
Source: BMC Cardiovasc Disord. 2024 Aug 2;24:401. doi: 10.1186/s12872-024-04062-z (PMC11295489; doi:10.1186/s12872-024-04062-z)

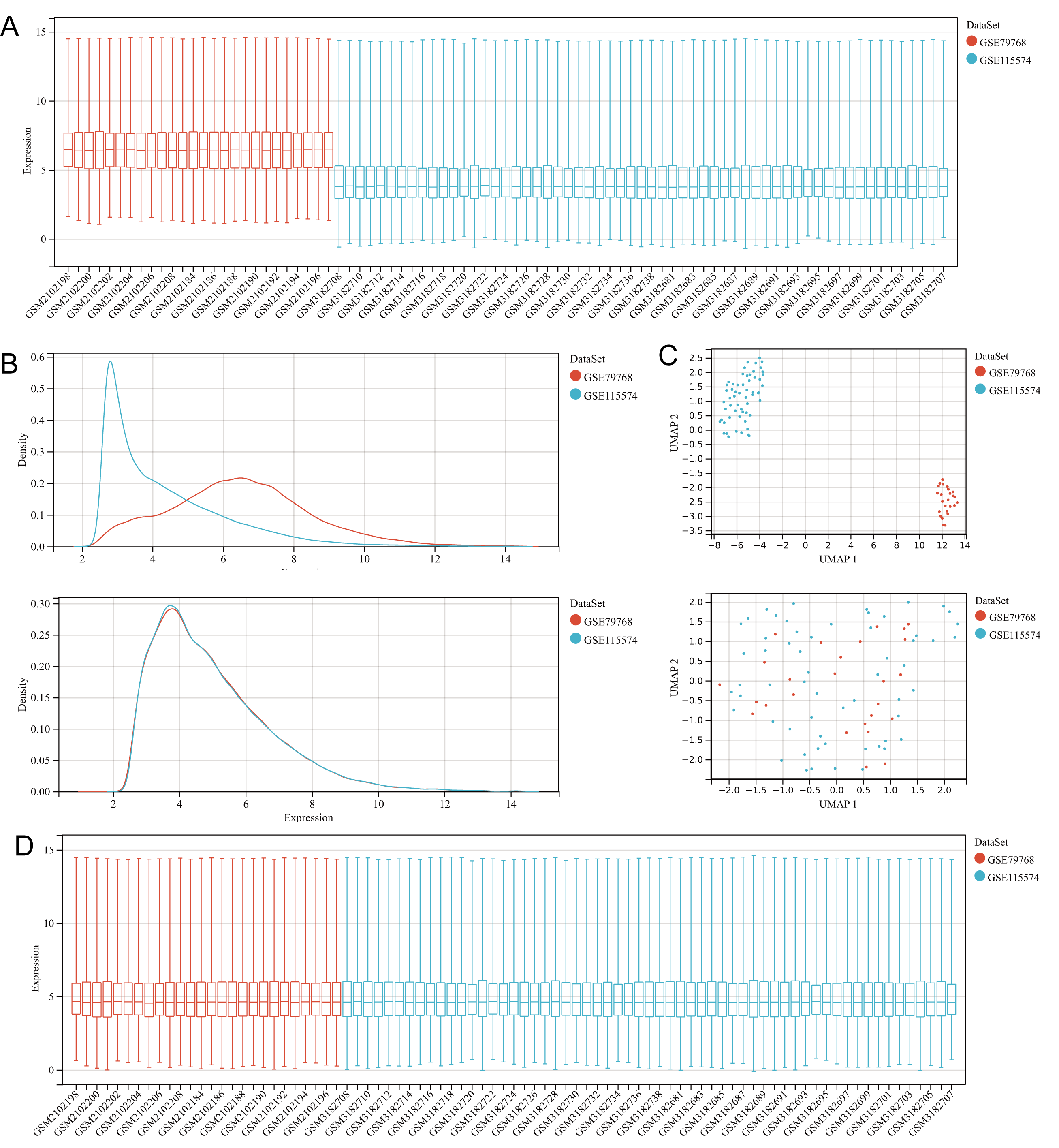

Supplement: Supplementary file 2 — Supplementary Material 2: Figure S1 Batch effect removal in merged GSE79768 and GSE115574 datasets for AF. (A) Box plot showing sample distribution before and (D) after batch effect removal. (B) Density plot illustrating sample distribution before (top) and after (bottom) batch effect removal. (C) UMAP plot demonstrates that samples from the two datasets cluster separately before batch effect removal (top) and intermingle after batch effect removal (bottom). [file 12872_2024_4062_MOESM2_ESM.png]
